# Navigating family planning and career development in plastic surgery

**DOI:** 10.1016/j.jpra.2026.04.001

**Published:** 2026-04-19

**Authors:** Simran K. Chandawarkar, Kaylee Leathers, Aleksandra Krajewski

**Affiliations:** a Northeast Ohio Medical University, 4209 OH-44, Rootstown, OH, USA; b Department of Plastic Surgery, The Ohio State University Wexner Medical Center, 915 Olentangy River Rd, Columbus, OH, USA; c Department of Plastic Surgery, Stony Brook University Medical Center, 4 Smith Haven Mall, Lake Grove NY, USA

**Keywords:** Professional planning, Family planning, Plastic surgery, Residency, Work-life balance, Wellness

## Abstract

Women in plastic surgery training face distinctive challenges in aligning reproductive health and family planning with the rigors of a demanding surgical career. With long training durations, competitive program cultures, and late entry into practice, childbearing during residency is often delayed or deprioritized. At the same time, plastic surgery has made measurable advances in gender representation, institutional policy, and cultural acceptance of pregnancy during training. No doubt, the progress that has already occurred over the must be explicitly recognized alongside ongoing gaps. To characterize these pressures, we conducted a narrative review of PubMed/MEDLINE and professional society resources (2000–2025) as well as a systematic PubMed search (2015–2025) that identified 11 quantitative studies specific to plastic surgery residents. Key domains included pregnancy timing, parental leave, fertility, disclosure, lactation, mentorship, and work-life balance. Across studies, many trainees reported postponing pregnancy and receiving minimal fertility counseling. Roughly 40% were dissatisfied with existing leave policies, and 20% delayed childbearing due to training demands. Small program sizes and intense competitiveness may heighten stigma around pregnancy, while insufficient institutional support, limited childcare, inadequate lactation facilities, and rigid scheduling amplify stress. Obstetric complications appear more common among surgical trainees compared to non-surgical peers. Although the American Board of Plastic Surgery allows up to 12 weeks of leave during residency, this is often insufficient to accommodate combined parental, medical, and family emergency needs. Importantly, recent literature also reflects increased program-level awareness and improving norms around parenthood, with growing attention to formal leave policies, lactation support, and trainee wellness. Addressing these systemic, cultural, and biological barriers requires multi-level action: reforming institutional policies, expanding support resources, fostering mentorship, and normalizing parenthood in training culture. This manuscript aims to support continued progress by pairing transparent discussion of persistent challenges with practical recommendations and examples of evolving best practices, with the goal of strengthening and not discouraging, future trainees’ interest in plastic surgery.

## Introduction

In all professions, the emotional toll of career-family choices impacts a woman’s mental well-being and anxiety. Plastic surgery is no exception. Despite a 10% increase in female residents in the last 8 years, the reproductive challenges created by years of school and research, 6+ years of intense training in a highly competitive practice environment, cannot be overlooked.^1^ Notably, plastic surgery has also experienced meaningful gains in gender representation and institutional attention to trainee well-being; however, alignment between training structure and reproductive health needs remains imperfect. For many women surgeons-in-training, this timeline overlaps with their ideal child-bearing years, heightening the stress caused by how building a family might impair them professionally.^2^^,^^3^ While several educators argue that these two goals are not mutually exclusive, balancing them is difficult for women plastic surgeons, generating guilt, anxiety, and helplessness. Many suffer silently or compromise one goal for the other. In parallel, an increasing body of literature and policy reform efforts suggests that pregnancy during training is becoming more visible, more discussed, and in many settings more supported than in prior decades. Although increased participation of women has helped draw attention to these conflicts, much more needs to be achieved. This paper identifies persistent struggles faced by women in plastic surgery and explores ways for them to achieve their professional aspirations and their reproductive goals without barriers, bias, or guilt. We additionally highlight progress to date and propose concrete, feasible strategies that training programs can implement to sustain continued improvement.

## Methods

The authors undertook both a targeted narrative review and a systematic review to synthesize evidence on how surgical training, particularly plastic surgery, intersects with pregnancy/parental leave, lactation, fertility/ART, disclosure, mentorship, work–life balance, and burnout. Searches were conducted in PubMed/MEDLINE and complemented by hand-searching reference lists and professional organization websites. For the narrative review, the working window was Jan 2000–Aug 2025 (English language), chosen pragmatically to capture contemporary policies and trainee experiences.

**Figure 1:**
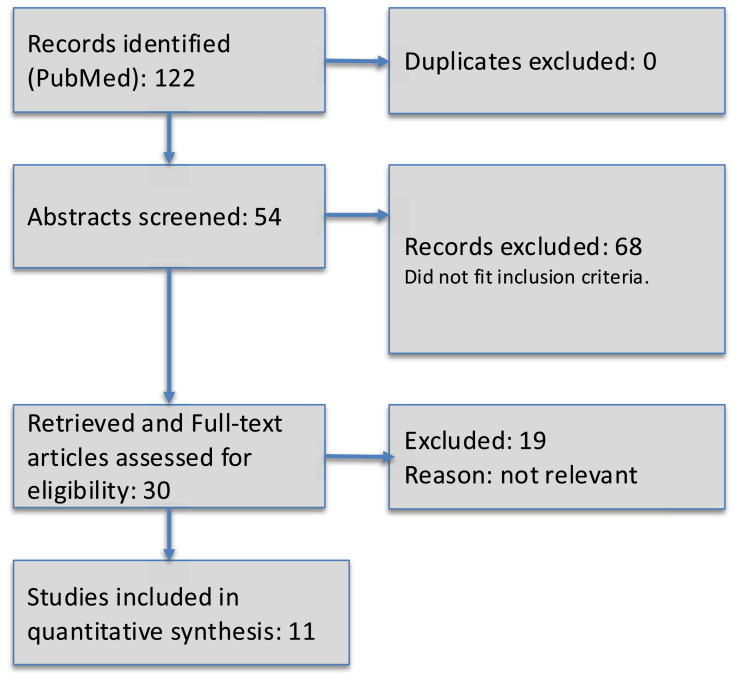
PRISMA diagram of included studies.

For a systematic review, a PubMed search was conducted in August 2025 for English-language articles published between 2005 and 2025 examining pregnancy, parental leave, fertility, or family planning among plastic surgery residents. Exact search terms were “(``plastic surgery''[Title/Abstract] OR ``plastic surgeon*''[Title/Abstract]) AND (``pregnancy''[Title/Abstract] OR ``parental leave''[Title/Abstract] OR ``fertility''[Title/Abstract] OR ``family planning''[Title/Abstract]) AND (``resident''[Title/Abstract] OR ``residency''[Title/Abstract]) AND (``survey''[Title/Abstract] OR ``cohort''[Title/Abstract] OR ``cross-sectional''[Title/Abstract])”. This search yielded 11 studies (Figure 1). Eligible studies included peer-reviewed full texts with quantitative data focused on plastic surgery trainees or mixed-surgical cohorts with extractable plastic-surgery subgroup data. Commentaries, conference abstracts, case reports or series with fewer than 10 participants, qualitative-only studies, studies of attendings or non-surgical specialties, and papers on wellness or burnout without a direct family-planning outcome were excluded.

## Results

Residency training poses significant challenges for female physicians seeking to balance reproductive health and career advancement. Studies have consistently shown that residents in surgical specialties face greater barriers than those in non-surgical fields (Table 1), reflecting both structural and cultural differences in how training programs handle maternity leave, fertility, and family planning.^4^, ^5^, ^6^, ^7^ However, the literature also indicates gradual improvements over time in formal policies, lactation support, and openness to parenthood during training, particularly after recent accreditation and institutional reforms. There is a difference between residents and their peers in other non-medical fields as well (Table 2). They were significantly less likely to take maternity leave compared to those in internal medicine or psychiatry, largely because the latter offered more flexible rotations and greater institutional support (Figure 3). In surgery, however, the demanding, skill-intensive nature of training fosters a perception that any time away from the operating room leads to loss of progress. These structural differences (summarized in Table 3) often mean that women in surgical specialties (compared to non-surgical fields) delay, alter, or even abandon family planning goals due to the professional risks associated with pregnancy and childcare during training.^5^^,^^8^^,^^9^ In the US, maternity leave & parental policies use different metrics for government employees vs. medical residents (Table 3). Even when formal leave policies exist, surgical residency programs typically provided the least allowable coverage.

**Table 1:** Age comparison: female resident graduates in medical vs. surgical residency programs.

Specialty	Average graduating age for females (years)	Typical length of training	Competitiveness
Internal medicine	28–30	3 no gap years	Only at high-profile programs
Pediatrics	28–30	3 no gap years	Only at high-profile programs
Family medicine	28–30	3 no gap years	Only at high-profile programs
Anesthesia	31–32	4 no gap years	Only at high-profile programs
Gynecology & obstetrics	31–32	4 no gap years	Only at high-profile programs
General surgery	32–35	5 possible gap years	Moderate to high
Plastic surgery	32–36	6 definite additional gap years	Very high, requires research output as medical student, gap years, research and papers during residency training.

**Table 2:** Summary of common themes and challenges across non-medical, high-stress professions.

Law	Finance	Tech	Entertainment
Women in their 30s–40 s reach senior roles	Senior roles often in late 30s–40s	Senior roles in mid-30 s–40s	Peak careers often before 40
Long hours, male-dominated firms, pressure to delay family planning	High stress, long hours, competition, financial pressures	Hustle culture, long hours, limited maternity leave	Youth bias, long hours, high competition, limited maternity leave
Mentorship programs, flexible work policies (though still rare), fertility preservation	Egg freezing, flexible work policies, women-focused networks	Company policies for parental leave, mentoring, more women in leadership	Maternity leave policies, advocacy for more behind-the-scenes roles

**Table 3:** Maternity leave & parental policy frameworks in the US: different for government employees vs. medical residents.

Category	Federal employee	State/private hospital employee	Surgical/plastic surgery resident
Paid maternity leave	12 weeks (paid)	Often 6–12 weeks (paid)	Often <6–12 weeks (mixed paid/unpaid)
Job protection	Yes (FEPLA/FMLA)	Yes (FMLA and individual policies)	Board requirements complicate leave
Lactation support	Generally provided	Varies by institution	Increasingly required by ACGME
Childcare support	Varies by employer	10%–33% offer childcare benefits	Rarely available
Guaranteed leave	Yes if eligible	Yes (policy dependent)	Yes but interpretations vary

In 2003, a survey in JAMA Surgery revealed that only half of surgical residency programs in the U.S. offered any paid maternity leave, and when offered, materinity leave was often between 4–6 weeks. Non-surgical residencies, in comparison, were far more likely to offer more generous leave and supportive parental accommodations. A study in 2019 revealed that the maternity leave requirements were still unchanged, and additionally, female surgical residents reported they felt hesitant to disclose pregnancies out of fear that it would harm their career progression or fellowship opportunities 9. This culture of silence and stigma compounds the emotional burden faced by trainees 4,5. Recent work suggests that disclosure norms and institutional supports are improving in many programs, but variability remains substantial across institutions and specialties.

The American College of Surgeons has also reported that residents who took time off for childbirth or reproductive health issues frequently encountered challenges in skill retention and progression. Because surgical training is highly dependent on hands-on technical experience, missing OR time is perceived as falling behind peers.^10^ Non-surgical fields, where progression is less skill-based, do not present the same risks. Compounding these issues, access to childcare, lactation rooms, or flexible schedules is often minimal in surgical departments, leaving many residents with little institutional support.^7^

**Figure 2:**
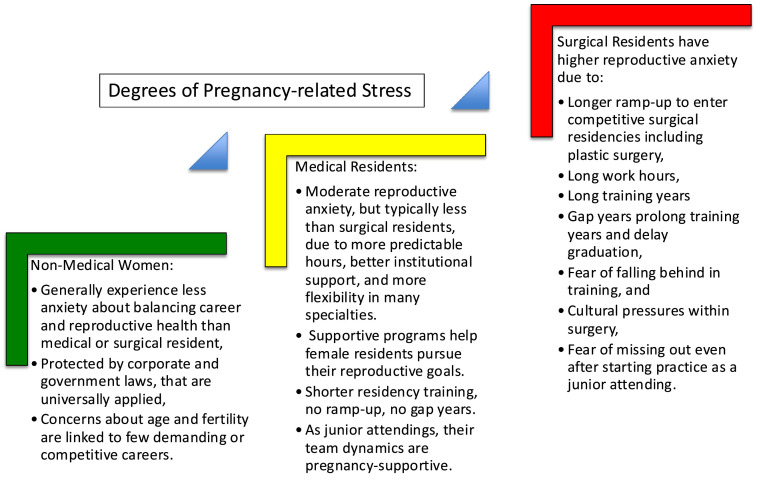
Why surgical residents face more pregnancy-related stress than medical and non-medical peers.

Across the 11 studies analyzed in the systematic review, the frequency of topics related to pregnancy and family planning is depicted in Figure 2. In general, 40% of survey studies mentioned residents are unhappy with their current parental leave policies, 20% reported postponing pregnancy due to residency, and less than half reported having adequate resources and counseling to make these decisions during residency. At the same time, multiple studies describe emerging program-level resources (policy formalization, lactation accommodation, wellness initiatives, and mentorship visibility) that may represent early markers of improving support.

**Figure 3:**
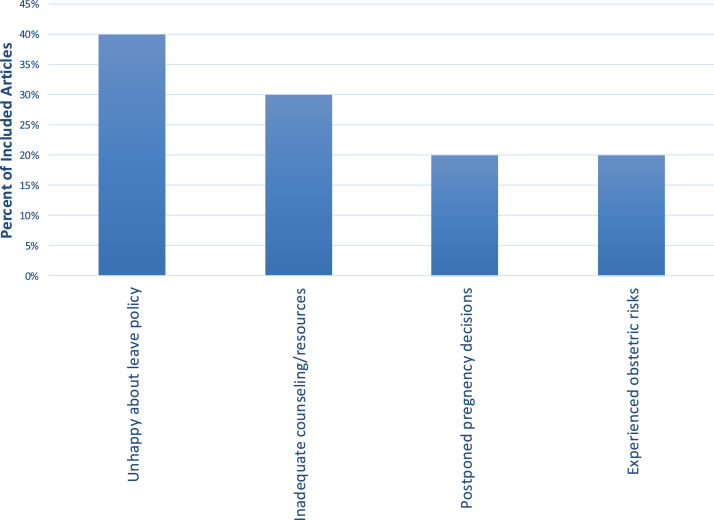
Themes from Included Cross-sectional Survey Articles of Residents.

*Progress and opportunities (2000*–*2025):* While this manuscript emphasizes persistent barriers, it is equally important to acknowledge the significant advances plastic surgery has made over the past two decades. Women’s representation in training has increased, institutional attention to resident wellness has expanded, and parental leave and lactation protections have become more visible in graduate medical education policy. Recent publications and program director surveys suggest increasing acceptance of pregnancy during training and greater willingness to discuss family planning openly. These developments reflect a specialty that is actively evolving. The remaining gaps described in this manuscript (leave adequacy, schedule flexibility, fertility counseling, lactation logistics, and childcare infrastructure) must be interpreted as opportunities for continued refinement rather than evidence that plastic surgery is an inhospitable field. In our view, transparent discussion paired with practical solutions strengthens recruitment and retention by demonstrating that plastic surgery is committed to supporting excellent surgeons across different life stages.

*Trends of changing perception of childbearing during residency training*: The papers revealed a 25-year trend to map changes in the attitudes and policies towards resident childbearing (Details in Appendix 1). Broadly, these trends are categorized in 4 tranches: 2003–2016 wherein literature was sparse, mostly older reviews or commentary summarizing limited research on pregnancy during residency training; 2017–2019 saw the first wave of empirical analytical survey studies on child-bearing during training plastic surgery and general surgery; 2020 where a systematic review consolidating early data highlighted ongoing pregnancy challenges for residents; and 2023–2025 which saw a renewed interest with policy-oriented reviews, cohort data, task force recommendations, and specialty-specific surveys coinciding with updates to ACGME and board policies.

*Challenges specific to plastic surgery:* While all surgical specialties present reproductive and family planning challenges, like vascular surgery and colorectal surgery, they are particularly magnified in plastic surgery, one of the most competitive and demanding training pathways.^11^^,^^12^ Plastic surgery residency attracts highly motivated trainees, many of whom take additional gap years in research or advanced degrees to strengthen their applications.^13^ As a result, plastic surgery residents often begin training later, and when combined with the 6–8 years of residency itself, women frequently graduate in their early-to-mid 30s-directly overlapping with the natural decline in fertility.

The residency is physically and emotionally taxing, characterized by long hours, high-stakes procedures, and pressure to continuously prove competence. Exhaustion itself becomes an unhealthy lifestyle factor that undermines reproductive health.^14^ The American Board of Plastic Surgery (ABPS) must be credited in that it does allow residents up to 12 weeks of leave (over the 6-year training) without having to extend their training. Some still consider this inadequate as it does not account for time needed for other life altering events during those 6 years such as illness or death in the family.^15^ Small program sizes also mean that maternity leave or extended absences place greater strain on colleagues and faculty, making accommodations less feasible.^16^ Unlike larger residencies, where schedules can be redistributed, plastic surgery programs often lack the bandwidth to provide meaningful flexibility.

The culture within plastic surgery further complicates family planning. Because the specialty is intensely competitive, many residents perceive pregnancy, childbirth, or childcare as a signal of reduced commitment to their career. This perception is reinforced by both peers and mentors, creating a stigma that discourages women from pursuing parenthood during training.^17^ Consequently, many trainees either delay having children, turn to assisted reproductive technologies (ART), or face significant burnout attempting to balance both professional and personal demands.^5^^,^^18^

*Reproductive timeline:* Female fertility is strongly age-dependent, with ovarian reserve and egg quality declining after the late twenties and accelerating after age 35.^19^^,^^20^ For women in plastic surgery, this decline aligns almost perfectly with the length of training. By the time many residents finish residency and fellowship, they are entering practice at ages when fertility is already diminished and the risk of complications increases.^21^ This biological reality creates profound anxiety for women who aspire to both motherhood and surgical careers.^18^ While ART and egg-freezing technologies provide options for delaying family building, they are costly, emotionally taxing, and far from guaranteed. Geographic variations in state-laws may impose further restrictions. Success rates vary with age and procedure, and the financial burden, often tens of thousands of dollars, is particularly difficult for residents already coping with medical school debt and modest trainee salaries.^22^ As such, ART offers only a partial solution, and its availability does not address the underlying structural problem: the misalignment between medical training timelines and the female reproductive window.^23^

**Figure 4:**
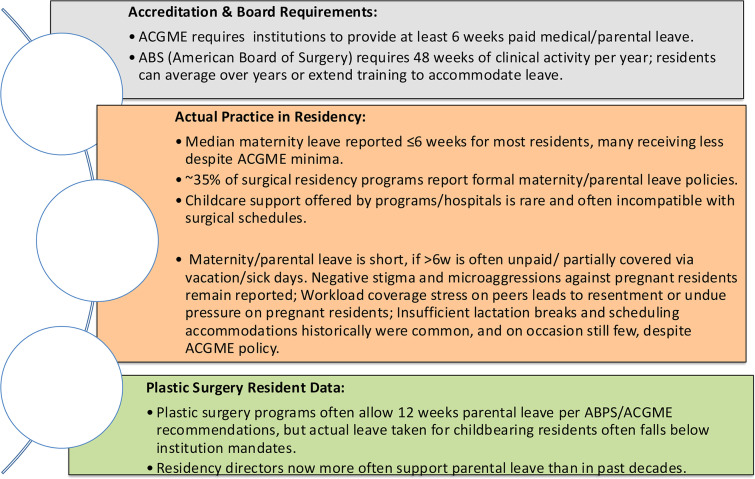
Gaps between regulatory requirements vs. actual practice.

*Support:* Despite requirements defined by regulatory bodies, there is a gap between the requirements and the actual practice (Figure 4). Despite the importance of professional and familial support in helping residents balance the competing demands of training and family life, institutional support varies significantly. Programs often lack clear parental leave policies, robust childcare options, or access to lactation facilities.^24^ Surveys through the Association of Program Directors in Surgery in 2023, of surgical residents revealed that: 67% did not have adequate time for pumping; 56% rarely had access to a lactation room; 69% of mothers reported a reduction in milk supply; 64% stated that the time constraints of residency shortened the total duration they breastfed and 59% of women did not feel comfortable asking to pump.^25^^,^^26^ Even when such resources exist, residents may hesitate to use them for fear of being perceived as less committed.^17^

Mentorship is another crucial but inconsistent support mechanism. Female residents who lack role models or mentors who have successfully navigated family planning during training often feel isolated in their struggles, especially during its long-drawn course.^27^ Conversely, supportive mentors can normalize discussions around fertility, pregnancy, and parenthood, reducing stigma and helping residents make informed decisions.^28^^,^^29^ Common mentorship approaches to advise residents are summarized in Appendix 2.

*Stereotypes and gender norms:* Gender stereotypes exacerbate the challenges faced by women in plastic surgery. In professions with a “masculinity contest culture” where long hours, unbroken availability, and stoicism are valorized, pregnancy and family responsibilities are known to be interpreted as incompatible with professional excellence.^30^^,^^31^ Women may fear that requesting accommodations, working part-time, or taking leave will be perceived as weakness or a lack of dedication. This perception discourages women from pursuing high-intensity surgical specialties or pressures them to delay family planning. Academic productivity analyses show that female physicians often experience early-career slowdowns associated with childbearing, though productivity tends to “catch up” later.^32^ Despite this, the stigma attached to motherhood during training persists, creating systemic inequities.^33^ A separate issue relating to perceptions of program directors (PDs) shed light on the variation within specialties and even between different specialties. PDs reported that: most did not believe that child-bearing and rearing during training, affected the resident’s wellness; their institution’s resident salary was sufficient to support 1 resident parent and 1 child; and only 20.6% confirmed there were fertility benefits for residents.^15^ In OB/GYN programs, 71% PDs self-reported being unaware the recommendations of the American College of Obstetricians and Gynecologists 2016 policy statement on parental leave.^34^ They reported offering shorter parental leaves than they themselves thought trainees should receive; 83% believed that becoming a parent negatively affected resident performance, and approximately 50% believed that having a child in residency decreased well-being.^34^

*The biological clock:* The intersection of plastic surgery’s long training pathway and women’s reproductive aging is particularly stark. Residents often begin training in their mid-to-late 20 s, and after additional years of residency, research, or fellowship, many do not complete their training until their early-to-mid 30 s. At this stage, natural fertility has already declined, and risks of complications such as miscarriage, gestational diabetes, and preeclampsia are increased.^35^ This overlap amplifies the pressure residents feel to either accelerate family planning during training or delay until after graduation, when risks are higher. Both options carry significant emotional and professional consequences, and few institutional policies adequately address this conflict.^36^

### Risks of later-in-life pregnancy

In general, 47% female residents more frequently delayed having children because of training, and 58% reported experiencing pregnancy/parenthood-based mistreatment.^37^ Pregnancy after age 35, often labeled a “geriatric pregnancy”, is associated with higher maternal and neonatal risks, including hypertension, preeclampsia, and genetic abnormalities. Recovery from childbirth can also take longer, delaying return to the physically demanding duties of residency.^38^ Notably, the prevalence of obstetric complications in female surgical residents was notably higher than that reported in large studies of women of similar socioeconomic privilege.^37^ In smaller programs, even brief absences can place strain on colleagues and jeopardize training timelines, creating feelings of guilt and self-blame among new mothers.^36^ At the same time, residents who postpone pregnancy entirely face other challenges: guilt over prioritizing career, societal scorn for delaying family, and stress surrounding diminished fertility.^18^ Many turn to egg freezing as a compromise, but again, outcomes are uncertain, and costs are prohibitive for trainees.

*Balancing post-partum recovery with career demands:* For those who do have children during training, support for postpartum recovery is minimal. Women are often expected to return quickly to full professional duties, which may involve long surgeries, overnight calls, and physically strenuous tasks.^39^ At home, they face the “second shift” of newborn care, with little institutional recognition of the toll this double workload takes. Burnout is common, yet rarely addressed, as maternal exhaustion is often normalized as part of motherhood rather than treated as a critical wellness issue.^40^

*Gender stereotypes in plastic surgery:* Since 2022, women have been the majority of matched residents in integrated plastic surgery programs in the USA.^41^ However, gender stereotypes and scrutiny of women who choose to have children, particularly in competitive programs still exist. The ranking of women's jobs has been historically justified on the basis of childbearing and domestic life whereas men are seemingly the best workers since they are ‘disembodied’ from any such responsibility.^42^^,^^43^ These structural inequities could potentially be factors affecting representation of women in surgery, especially in higher academic positions, such as program chairs and directors, with female representation of 9.2% and 13.1%, respectively, and gender disparity in industry payments.^44^ Systemic barriers before, during, and after plastic surgery residency training seem to influence the representation of women in plastic surgery at all levels which could potentially discourage many women from either entering the field or advancing within the profession into leadership roles.^29^

*Variation by country:* Residency support for new parents varies widely across countries, shaped by national labor laws, healthcare systems, and cultural attitudes toward work-life balance. In many European nations, mandated parental leave policies and subsidized childcare reduce the conflicts between residency training and family planning. For example, in Denmark, every parent is entitled to 24 weeks of maternity leave (4 weeks before birth).^45^ Comparison of maternity benefits in the US vs. other countries in both, US employees vs. other countries and specifically hospital employees and surgical residents in the US vs. other countries are shown in Table 4, Table 5. Additionally, these weeks are transferable between two parents to best accommodate the lifestyle of each family on an individualized basis. Perception of support is also widely different between residents in the US and other countries (Appendix 3). This could be partly because U.S. programs often lack standardized policies, many times leaving decisions up to individual departments and creating wide variability in support. The percentage of women holding positions as chief/chair physicians is 49% in Denmark vs. 25% in the USA.^46^ Such international comparisons highlight that the challenges faced by female surgical residents are not inevitable, but rather products of institutional and cultural choices.^47^

**Table 4:** Comparison of maternity benefits in the US vs. other countries in both, US employees vs. other countries (upper table.

Country/region	Maternity leave	Paid?	Parental leave
USA (most employees)	12 weeks (unpaid)	No	Varies
Federal USA employees	12 weeks	Yes	Yes (policy dependent)
Canada	16 weeks	Yes	Yes (shared)
Europe	14–20 weeks	Yes	Yes
Japan	14 weeks	Yes	Yes
India	26 weeks	Yes	Yes

**Table 5:** Comparison of maternity benefits in hospital employees and surgical residents in the US vs. other countries.

Domain	Hospital employees (US)	Surgical & plastic surgery residents (US)	Employees & all residents in all other countries
Paid maternity leave	6–12+ weeks common	6 weeks minimum (ACGME)	14–26+ weeks
Leave funding	Employer/state	Program/hospital GME	National social insurance
Job protection	FMLA + Contracts	Board-dependent	Statutory
Lactation support	Often standardized	Now required	Standardized
Schedule modification	Common	Variable	Common
Childcare support	Increasingly offered	Rare	Often subsidized
Cultural stigma	Low	Moderate	Low

## Discussion

Our study highlights the difficulties female residents face and the guilt and shame that come from their choices. Addressing these in a meaningful way requires a multipronged approach that includes several dimensions. We also emphasize that many programs and institutions are actively improving support structures, and the specialty has made substantial progress in gender equity and trainee wellness; the aim of this section is to identify feasible next steps to sustain that momentum.

Seeking balance seems difficult, if not impossible, for most women residents seeking to have a family, and making conscious choices about career paths and family planning often yield to compromises. Strategies for balancing career and reproductive goals must include:

*Institutional support:* Steps to ease the burden of balancing family life with a career in plastic surgery might need to include flexible scheduling options, part-time training opportunities, and longer accommodations for maternity leave. Addition of mentorship programs that emphasize work-life balance and provide real-life examples of female surgeons who have successfully navigated both motherhood and professional achievement could help generate confidence in new moms that the institution ‘has their back’. It also normalizes the concurrent pursuit of reproductive and career goals.

Addressing gender bias and creating a more inclusive culture in surgery is essential for making the profession more accessible to women, particularly when it comes to reproductive health. By actively fostering an environment where both male and female surgeons share caregiving responsibilities, surgical programs can reduce the stigma surrounding maternity leave and childbearing. This requires creating a culture where both men and women are supported in balancing work and family. Appendix 4 summarizes the elements that some of the most supportive surgical programs provided.

*Proposed solutions:* To strengthen the manuscript’s actionable value, we propose the following practical, implementable program-level recommendations (adaptable across program sizes):

1. Standardize written parental leave policies with transparent coverage plans (e.g., predefined “float” coverage, elective redistribution, cross-rotation support).
2. Protect lactation time operationally (scheduled blocks) and site lactation rooms within practical proximity to OR/clinics to reduce time burden.
3. Provide structured fertility and family-planning counseling early in residency (including ART coverage clarity and referral pathways).
4. Implement “return-to-work” ramp rotations post-leave (lighter call, clinic-heavy blocks) to support safe re-entry and reduce burnout.
5. Formalize mentorship visibility by pairing resident parents (or those planning families) with faculty mentors who have navigated pregnancy/parenthood in surgery.
6. Where childcare capacity is limited, offer bridge solutions (subsidies, priority waitlists, emergency backup care partnerships) to reduce attrition pressure.

Results also showed that several pregnancy-related issues are stressful, including delayed family planning, fertility struggles, and the pressure of post-partum demands, balancing demanding careers with motherhood. In response, the institution must make available easy-to-access mental health resources to address health implications via promoting discussions on stress, anxiety, or even depression among women in these professions, and if needed, therapy and treatment. This could significantly alleviate the risks of burnout and their ability to balance work and family, and their biological concerns. In a study conducted at Johns Hopkins University School of Medicine, male and female residents in various specialties were surveyed regarding their experiences with family leave. The results showed that programs that offered paid parental leave, flexible scheduling, and shared responsibility for family caregiving (with equal participation from both men and women) saw higher job satisfaction and retention rates for female surgeons. This culture of shared responsibility and mutual support helped eliminate some of the societal and professional biases that hinder women from pursuing both career and family goals.

*Societal help:* A vital link to offset the poor availability of affordable childcare, especially for extended work hours and on-call days. This commonly becomes a compromise with either one parent working from home, or a family member/neighbor/friend that fills in as an ad hoc child caregiver. More accessible childcare or financial assistance by institutions will help relieve the relationship strain this can cause.

*Mentorship and networking:* Women mentors who have successfully navigated the challenges of balancing a surgical career with parenthood can offer valuable pointers to younger mentees (also summarized in Appendix 2).^48^^,^^49^ These include strategies to minimize the career impact of maternity leave, navigate career gaps, and manage work-life stress. Similarly, a virtual web-based network of other residents experiencing similar challenges could provide emotional support and practical insights into managing family and career aspirations.^50^

*Collaborative work arrangements:* In many highly competitive non-medical professions, women also face unique challenges when it comes to balancing career advancement with reproductive goals due to the demanding nature of the work, long hours, high levels of responsibility, and the desire to delay family planning in favor of establishing professional success. In sharp contrast to the medical fields, these professions are much more adept at creating collaborative work arrangements than plastic surgery. Some of the largest USA-based corporations are the leaders in parental benefits, supporting new parents to match their individual needs, where medicine is starting to fall behind. Women surgeons starting practice could use these templates to balance their family and career goals by seeking out collaborative work arrangements, job-sharing positions, part-time roles, or non-traditional practice structures, such as group practices, which allow greater flexibility in scheduling.

*Personal self-care and wellness:* It is critical for surgeons to prioritize their physical and mental health during their careers. Women who seek to balance reproductive goals with a demanding career in plastic surgery should make time for self-care, including exercise, relaxation, and therapy when necessary. Ensuring a healthy work-life balance is essential not only for personal well-being but also for professional success. Encouraging self-care and providing wellness programs within institutions can foster an environment where trainees and practitioners are supported in maintaining both their personal and professional lives.

It can be argued that a lack of structured studies would be much more instructive to make improvements, and this study simply identifies the problems and does not provide validated solutions. This criticism is fair. However it must be noted that there are very few if any analytical studies that have longitudinally studied this subject to be able to make solid, validated recommendations. Further, as one can see in the trends, these changes over the past 25 years have been in response to opinion rather than data. We therefore frame our recommendations as feasible, evidence-informed steps grounded in the available survey and policy literature, while emphasizing the need for prospective, outcomes-based studies (e.g., retention, operative competency metrics, well-being, and patient safety) to evaluate the impact of specific interventions. This study hopes to create awareness - the first step towards seeking to change ones outlook from a fixed mindset (‘this is how it always was’ or 'we did it so you can too’) - to a more robust forward facing growth mindset.

## Conclusions

Plastic surgery has made substantial and measurable progress in advancing gender equity, parental leave policies, and cultural acceptance of pregnancy during training. The specialty today differs markedly from that of two decades ago, and these advances deserve recognition. At the same time, continued improvement remains possible and necessary. Our intention is not to portray plastic surgery as inhospitable, but rather to highlight opportunities for further structural refinement that will ensure the field continues to attract, retain, and empower talented surgeons of all genders. Transparency about challenges, paired with an acknowledgment of progress and practical solutions, will strengthen, not diminish, the appeal of plastic surgery to future generations.

## Funding

No funding was received for this project.

## Ethics statement

All ethical considerations were taken. No human or animal subjects were part of this work.

## Declaration of competing interest

Aleksandra Krajewski, M.D. is a cMedical Consultant for Avita.

## Appendix B Supplementary materials

Image, application 1

Image, application 2

Image, application 3

Image, application 4
