# Supplementary material for: Navigating family planning and career development in plastic surgery
Source: JPRAS Open. 2026 Apr 19;50:221–33. doi: 10.1016/j.jpra.2026.04.001 (PMC13197714; doi:10.1016/j.jpra.2026.04.001)
Supplement: Supplementary file 1 [file mmc1.docx]

**Appendix # 1 Trends in Resident-Maternity Outlook between 2000-2025, Mapped with Key Citations**

To simplify the trends, we have categorized them into the following groups:

**2000-2005: Problem Identification:** First set of studies documenting: Discouragement of pregnancy during surgical training**;** Delayed childbearing and infertility**;** Lack of formal leave policies**.** Early surveys showed leave durations often ≤2–4 weeks, typically deducted from vacation. Plastic surgery residents reported amplified stigma due to small program size and call coverage constraints. Representative literature themes: Pregnancy treated as a personal choice with professional consequences; absence of institutional responsibility.

**2006-2015: Awareness Without Enforcement.** National surveys in surgery revealed: Persistent lack of standardized maternity policies; Continued pressure to maintain full operative workload during pregnancy; “Wellness” entered academic language, but pregnancy was not yet framed as an occupational health issue.

**2016-2019: Data-Driven Advocacy.** Large multi-institutional studies showed: Higher complication rates during pregnancy among surgical trainees; Fear of negative evaluations and career repercussions. Plastic surgery-specific publications highlighted: Underreporting of pregnancy; Informal accommodations dependent on program leadership.

**2020-2022: Structural Reform:** ACGME mandate establishes: Minimum 6 weeks paid parental/medical leave; Required lactation space and protected time; ABS policy updates allow averaging of training time across years and avoidance of automatic training extension. The significance of this was the shift from ‘cultural permission’ to ‘regulatory obligation’.

**2023-2025: Measurable Cultural Shift:** Program director surveys (including plastic surgery): Increasing acceptance of pregnancy during residency**;** Neutral or positive views on resident performance**;** Documented increase in pregnancies during plastic surgery training compared with pre-2015 cohorts. Persistent findings: Residents still return to work earlier than hospital employees and international counterparts.

**Mapped Residency + Pregnancy Articles (PubMed Search)**

| **Year** | **Citation (APA/MLA style)** | **Specialty** | **Study Type** |
| --- | --- | --- | --- |
| 1999 | Levinson AJ. Pregnancy during residency training: effect on surgical experience. Am J Obstet Gynecol. 1999; (Study explored surgical volume in obstetrics & gynecology residents during pregnancy). | Obstetrics & Gynecology | Analytical (survey + database) |
| 2005 | Pregnancy Among Women Surgeons: Trends Over Time. JAMA Surg. 2005; (Examined pregnancy timing, stigma, and attitudes among women in surgical fields). | General Surgery / Multiple | Analytical |
| 2010 | Resident attrition study: Pregnancy-related attrition in general surgery. J Surg Educ. 2010; (Retrospective analysis of attrition rates and childrearing). | General Surgery | Analytical |
| 2017 | Humphries LS, Butz DR, Song DH. Pregnancy and the Plastic Surgery Resident. Plast Reconstr Surg. 2017;139(1):253-255. | Plastic Surgery | Analytical (director survey) |
| 2018 | Rangel EL, Smink D, Castillo-Angeles M, et al. Pregnancy and Motherhood During Surgical Training. JAMA Surg. 2018;153(7):644-652. | General Surgery | Analytical (national survey) |
| 2019 | The Pregnant Female Surgical Resident (survey of recent pregnancies and perceptions in general surgery). Am Surg. 2019; (Reports maternity leave lengths, exam performance aspirations, etc.). | General Surgery | Analytical (survey) |
| 2020 | Pregnancy During Surgical Training: Are Residency Programs Truly Supporting Their Trainees? Ann Surg. 2020; (Survey of pregnancy loss, leave, schedule adjustments). | Surgical Residents | Analytical (cross-sectional survey) |
| 2022 | Ward CM, Wong CA, Prince JM, et al. Evolution in Parental Leave Policies: Plastic Surgery and Obstetrics/Gynecology Lead the Way. Plast Reconstr Surg. 2022;(Trends in parental leave across specialties). | Multiple Specialties incl. Plast Surg | Analytical (policy comparison) |
| 2024 | Bernal I, Moon SL, Hotta M, Newman MI. Residents’ Perspectives of Pregnancy and Growing a Family During Surgical Training: A Review of the Literature. Cureus. 2024; (Systematic review of surgical resident pregnancy experiences). | Surgery / Multiple | Review (literature synthesis) |
