# Supplementary material for: Navigating family planning and career development in plastic surgery
Source: JPRAS Open. 2026 Apr 19;50:221–33. doi: 10.1016/j.jpra.2026.04.001 (PMC13197714; doi:10.1016/j.jpra.2026.04.001)
Supplement: Supplementary file 2 [file mmc2.docx]

Appendix 2: Mentorship approaches and associated narratives that residents could benefit from.

**Understand that ‘You are not Alone’**

Narratives:

- It’s easy to feel like you are missing your window. But trust me, there is no ‘perfect time.’ Life, including fertility, has its own rhythm, and you need to adapt. Family planning doesn’t have to happen according to a textbook timeline."
- Avoid comparisons with others’ timelines or choices, whether regarding career milestones or family planning.
- Embrace your individual journey and make decisions that align with your values and goals.
- Having children does not diminish your ability to be a world-class surgeon. But the truth is, you may have to prioritize family over work at certain points in your life. When you choose to have children, there will be times when family comes first, and that’s okay. That doesn’t mean your career is over. The challenge is figuring out how to integrate these parts of your life without sacrificing your happiness or well-being;

**Prioritize Self-Care and Mental Health**

- Mental health should be a priority. Seek help if you need it.
- Don’t shy away from taking breaks or asking for support when you’re feeling overwhelmed.
- Develop a healthy work-life balance as early as possible.

**Flexibility**

- Ask for flexibility in your work schedule if needed (for family planning, childcare, or mental health reasons).
- Set clear boundaries about your availability when it comes to work, especially if you need time for personal matters.

**Lean on Your Support System**

- Cultivate a strong support network—surround yourself with people who understand the demands of your profession and your personal goals.
- Mentorship is invaluable. Find mentors who can offer guidance not just on surgery, but on life balance.

**Learn to Navigate Gender Bias**

- Recognize and confront gender bias when it happens.
- Trust your abilities and don’t second-guess yourself because of your gender.
- Stay confident in your skills and remember, your presence in this field is well-deserved.

**Family Planning Takes Patience—But It’s Possible**

- Take your time with family planning. It’s okay to delay starting a family if you’re focused on your career or feel like the timing isn’t right.
- Explore options like egg freezing if you’re concerned about fertility and want to safeguard your future choices.

**Don't Let Guilt Overwhelm You**

- Being a female surgeon means you’ll sometimes feel like you have to be superwoman—able to handle everything perfectly. This can lead to immense guilt, especially when it comes to reproductive goals.
- Let go of guilt. You’re doing your best, and that’s enough.
- Seek balance, not perfection—you don’t have to be perfect in all aspects of life to succeed.

**Think Long-Term**

- Stay focused on your long-term goals. Early years in surgery may be challenging, but they lead to greater flexibility later on.
- Plan ahead for future work-life balance when you reach a more senior stage in your career.

**Celebrate Your Achievements—No Matter How Small**

- Celebrate the small wins—don’t wait for the big moments to recognize your success.
- Acknowledge your achievements and give yourself credit for the hard work you put in.

**Explore Fertility Preservation Options Early**

- Egg freezing is a good option to consider if you’re planning on delaying starting a family due to the demands of residency or fellowship.
- Understand that fertility can be a concern after a certain age, particularly in high-stress fields, and proactive planning can help alleviate future worries.

**Realistic View of Work-Life Balance**

- During residency and fellowships, expect periods of imbalance. You’ll work long hours, and family time might be limited. But it’s temporary, and once you gain more control over your schedule, balance becomes more achievable.
- As you progress in your career, you’ll have more control over your schedule, which can help you adjust work-life balance to accommodate your family planning goals.

**Be Strategic About Your Career and Family Planning**

- Set clear boundaries early on regarding work commitments, especially if you plan to have children during your training or early career.
- Plan strategically—know when to push forward with your career and when to slow down to focus on family goals.

**Don’t Let Fear of Losing Opportunities Stop You**

- Don’t be afraid that family planning will derail your career. Many women find that motherhood enhances their ability to manage time, prioritize, and grow their leadership skills.
- Make decisions based on your goals, not on fears about missing opportunities. You can build a successful career and have a fulfilling personal life—just at different times or in different ways.
