# Supplementary material for: Navigating family planning and career development in plastic surgery
Source: JPRAS Open. 2026 Apr 19;50:221–33. doi: 10.1016/j.jpra.2026.04.001 (PMC13197714; doi:10.1016/j.jpra.2026.04.001)
Supplement: Supplementary file 3 [file mmc3.docx]

**Appendix 3: Comparison of maternity benefits and their perceptions between the US and other countries.**

United States

- Parental Leave: The U.S. does not have a federally mandated paid parental leave for residents, and the availability of paid maternity leave is limited. Many residency programs offer between 6-8 weeks of maternity leave, but this is often unpaid or only partially paid.
- Flexibility and Childcare: Childcare support is limited in the U.S., and on-site daycare is rare. Many residents face significant financial strain and logistical challenges when it comes to childcare. Some institutions may offer limited flexibility, but this is not universal, especially in surgical specialties.
- Support Perception: U.S. residents often report that the support offered is insufficient. A 2020 survey published in JAMA Surgery found that only 50% of surgical residency programs provided paid maternity leave, and many residents in surgical fields do not feel adequately supported after having a baby.

The following 3 countries have a nationalized health system. All residents and physicians are salaried employees of the government and policies that apply to non-medical employees apply to them as well. Secondly these countries do not typically operate on a for-profit model. Hence the economic ‘cost’ of a pregnancy or of maternity leave is not seen to affect the bottom-line. It is seen as a women’s right, instead.

United Kingdom

- Parental Leave: The U.K. has more robust parental leave policies compared to the U.S. New mothers are entitled to up to 52 weeks of maternity leave, with 39 weeks being paid. Partners are also entitled to shared parental leave.
- Flexibility and Childcare: The U.K. offers more flexibility in terms of part-time work or adjusted hours, particularly for new parents. Additionally, some hospitals provide on-site childcare services, though these may still be limited.
- Support Perception: While maternity leave policies in the U.K. are generally seen as supportive, challenges remain regarding the affordability and accessibility of childcare. There is also variability in how flexible work arrangements are, with some specialties being more supportive than others.

Canada

- Parental Leave: Canada provides one of the most generous parental leave policies globally. New mothers are entitled to up to 18 months of leave, with a portion of it being paid at a rate of 55% of their salary (up to a maximum). Partners are also eligible for parental leave.
- Flexibility and Childcare: Childcare subsidies are available in many provinces, though the availability of on-site childcare varies. Flexibility in work hours and accommodations for breastfeeding or pumping are also more common in Canada, particularly in non-surgical specialties.
- Support Perception: Canadian residents generally report feeling more supported than their counterparts in the U.S., though surgical specialties still present challenges in terms of work hours and flexibility.

Australia

- Parental Leave: Australia offers up to 18 weeks of paid parental leave at the national minimum wage for eligible parents. There are also provisions for shared parental leave.
- Flexibility and Childcare: Many Australian hospitals offer flexible working hours and on-site childcare, though this is more common in urban centers. Programs vary widely depending on the location and specialty.
- Support Perception: Overall, Australia’s policies tend to be supportive of new parents, but challenges still exist, particularly for residents in high-demand surgical specialties who may have fewer flexible options.
