# Supplementary material for: Navigating family planning and career development in plastic surgery
Source: JPRAS Open. 2026 Apr 19;50:221–33. doi: 10.1016/j.jpra.2026.04.001 (PMC13197714; doi:10.1016/j.jpra.2026.04.001)
Supplement: Supplementary file 4 [file mmc4.docx]

**Appendix #4 Residency Programs & Institutions Known to Be Supportive of Pregnancy/Parenthood**

1. **Massachusetts General Hospital (MGH)**

**General Surgery & Internal Medicine**

General Surgery Residency (MGH): Explicit departmental support for pregnancy, parental leave, and caregiver responsibilities. Their policies: Celebrate childbirth as a positive event and support both birthing and non-birthing parents; Ensure workplace accommodations for pregnancy and early parenthood; Protect against discrimination, bullying, or punitive scheduling related to family planning.

Internal Medicine/DOM Residency (Mass General Brigham): Up to 8 weeks of paid parental leave for all new parents, with the possibility of combining with vacation for up to ~12 weeks total; Allows flexible scheduling of parental leave during training, arrangements for prenatal appointments, and a “softer landing” period on lighter rotations post-leave; Leave does not automatically require residency extension if competency requirements are met.

Institution-wide benefits include: Up to 12 weeks of leave via Mass General Brigham policy, available for both birth and adoptive parents; Access to lactation rooms and pumping support, coordinated through organizational resources like “Mothers’ Corner”.

**Data Source:** Institutional leave policies (MGH DOM and surgical residency manuals) and organizational parental leave frameworks.

1. **University Hospitals – General Surgery Residency (Cleveland, OH)**
   1. General Surgery Residency Program has a written parental leave policy that explicitly states: Residency should not preclude having children; The program will support both birthing and non-birthing residents; Reasonable pregnancy-related accommodations (schedule adjustments, rotation modifications, call coverage considerations) are part of the policy.**Data Source:** Official residency document: General Surgery Division parental leave policy.
2. **Boston’s Teaching Hospitals & AHC-Style Programs: (**Examples are institutional, not always specialty-specific)
   1. Several major academic health centers (AHCs)-particularly in the Northeast-have onsite childcare and institutional infrastructure that benefits resident parents.
   2. A 2022 study found that ~62% of AHCs offer onsite childcare, which is a substantial family-friendly resource for trainees with infants or young children.
   3. **Institutions often cited (by residents and in housing policy surveys):** Massachusetts General Hospital / Mass General Brigham; Boston Children’s Hospital / Harvard-affiliated AHCs; University Hospitals Cleveland; Others with strong onsite childcare or lactation facilities based on institutional policy audits and resident reports.

**Specialty Specific Examples:**

**Dermatology Residencies with Structured Parental Leave.** A study of dermatology residency programs revealed: Many programs provide designated new parent leave ranging from ~4-14 weeks; Some do not require residents to make up time to sit Board examinations; Access to breastfeeding support may vary by institution, but most offer designated pumping spaces -though satisfaction between residents and program directors may differ. **Data Source** JAMA Dermatology survey of dermatology residency directors and residents.

**Pediatrics & Non-Surgical Specialties Tend to Have More Supportive Cultures:** A multi-specialty nationwide survey indicated that pediatric residents reported higher perceived support for parental leave, longer leave taken, and better perceptions of capability returning from leave compared with surgical trainees. While not tied to a specific program name, pediatrics residency environments often have more supportive cultural norms and leave practices than surgical training programs in general.

**Family Medicine Residencies with Varying Leave Policies.** Family medicine residencies demonstrate wide variability in parental leave offerings: Paid maternity leave can range from 0 to more than 12 weeks. Larger programs tended to provide more generous paid parental leave. Some programs offer at-home elective rotations for new parents to facilitate continuity of training without burdensome service obligations. **Examples of Supportive Practices include:** Use of lighter rotations or research electives during pregnancy; Institutional encouragement of protected leave without negative career impact.

**What “Supportive” Typically Includes in Practice**

Across these programs and institutional policies, the most commonly cited supportive features are:

| **Support Feature** | **Meaning / Example** |
| --- | --- |
| Paid parental leave beyond minimum | 8–12 weeks paid in some internal medicine and surgery–affiliated programs (Mass General Brigham) |
| Flexible scheduling for prenatal care | Program-specific accommodations for prenatal appointments and duty modifications |
| Lactation support | On-site lactation rooms and scheduled breaks offered at many major academic centers |
| No mandatory training extension for leave | Interpretations where board requirements can be met without extending training if competencies are achieved |
| Written formal residency policies | Explicit language supporting pregnancy, family leave, and schedule flexibility (University Hospitals) |
| Childcare infrastructure | On-site childcare at many academic health centers supports ongoing parenting duties |

**Published Research:** National surveys and institutional policy audits highlight variability and examples of supportive programs. **Institutional Parental Leave Documents from** formal policies from Mass General Brigham/ MGH and University Hospitals provide concrete examples of supportive policies.  The American College of Surgeons supports at least 6-12 weeks parental leave and reasonable workplace accommodations for trainees.  **Multi-Specialty Surveys** comprise Studies comparing specialties show residents in pediatrics and internal medicine often perceive more support than those in surgical disciplines.
